# Anthropause on audio: The effects of the COVID-19 pandemic on church bell ringing and associated soundscapes in New South Wales (Australia)a)

**DOI:** 10.1121/10.0002451

**Published:** 2020-11-25

**Authors:** Murray Parker, Dirk HR Spennemann

**Affiliations:** 1 School of Environmental Sciences, Charles Sturt University, P.O. Box 789, Albury New South Wales 2640, Australia; 2 Institute for Land, Water and Society, Charles Sturt University, P.O. Box 789, Albury New South Wales 2640, Australia

## Abstract

The use of religious bells as symbolism and ritual is prevalent in many faiths worldwide. However, the sound of bells emanating from churches is by nature not exclusive to the church, as these sounds can effectively become part of the “public domain.” The value of church bell ringing can therefore be attributed to the church community and clergy as well as the wider community. Cessation of these sounds affects not only the soundscape of the area, but the people who place value on these sounds or soundscapes. Data are presented from a previous survey from 2018 investigating church bell practices in New South Wales (Australia) and compared to the current practice of bell ringing, which has been heavily influenced by regulations introduced due to the COVID-19 pandemic.

## I. INTRODUCTION

The emergence of COVID-19, the coronavirus disease caused by the severe acute respiratory syndrome coronavirus 2 (SARS-CoV-2) (WHO, 2020), in January 2020, and its rapid development into a global pandemic, have acted as a cross-sectorial disruptor. Day-to-day processes of commerce, transportation, recreation, and social interaction were disrupted and temporarily suspended or curtailed, especially during the imposed periods of lockdown. These disruptions of daily processes resulted in changes to soundscapes in particular to those of urban areas. This is reflected in a reduction of ambient traffic noises [e.g., Hastreiter (2020)], but also in a reduction of community-wide signal sounds, such as church bells (Eggington, 2020). Building on research conducted during the pre-pandemic period (Parker and Spennemann, 2020), we will examine the impact of implemented public health measures on religious soundscapes in New South Wales (NSW) (Australia).

## II. STATUS OF CHURCH BELL RINGING PRE-COVID-19

We undertook a survey in 2018 to investigate past and current trends of church bell ringing practices across NSW, looking at the six most populous denominations of the Christian faith in 2016 (Australian Bureau of Statistics, 2017): Roman Catholic, Anglican, Uniting Church, Eastern Orthodox and Presbyterian and Reformed. Our original study used a mixed methods approach to solicit information pertaining to the presence/nature of church bells, frequency/occasions on which they were rung, augmented with opinions and value judgments regarding bell ringing. While full results of the study are reported elsewhere (Parker, 2018; Parker and Spennemann, 2020), we wish to highlight important results from this snapshot of 2018, to make a direct comparison to practices currently occurring due to imposed restrictions as a result of the COVID-19 pandemic.

A large proportion of Orthodox (91%), Catholic (78%), and Anglican churches (76%) reported bells on church premises,^1^ such as a tower bell, altar/Sanctus bell, sacristy bell, recorded bell, or other bells. Churches from Orthodox and Anglican denominations had the highest representation across the majority of bell ringing activities, and a high proportion of churches from all denominations rang bells for regular services. Weddings saw a high frequency of bell use for all denominations, as did funerals for Anglican, Catholic, and Orthodox churches. The majority of Catholic and Orthodox churches rang their bells more than twice per week, with Anglican churches ringing either similarly or once a week.^1^ The results from 2018 show that bell ringing at that time was popular in many churches of Anglican, Catholic, and Orthodox faith, with a majority of these churches having bells on their church premises, ringing them regularly, and ringing them for both sacred and secular functions. They also show that while churches from Presbyterian and Uniting Church faiths are generally underrepresented with respect to bells on their premises, a large number of churches who have bells rang them for regular services or for yearly events.

## III. TIMELINE OF GOVERNMENT-IMPOSED REGULATION IN NSW UNTIL MID-AUGUST 2020

**FIG. 1.**
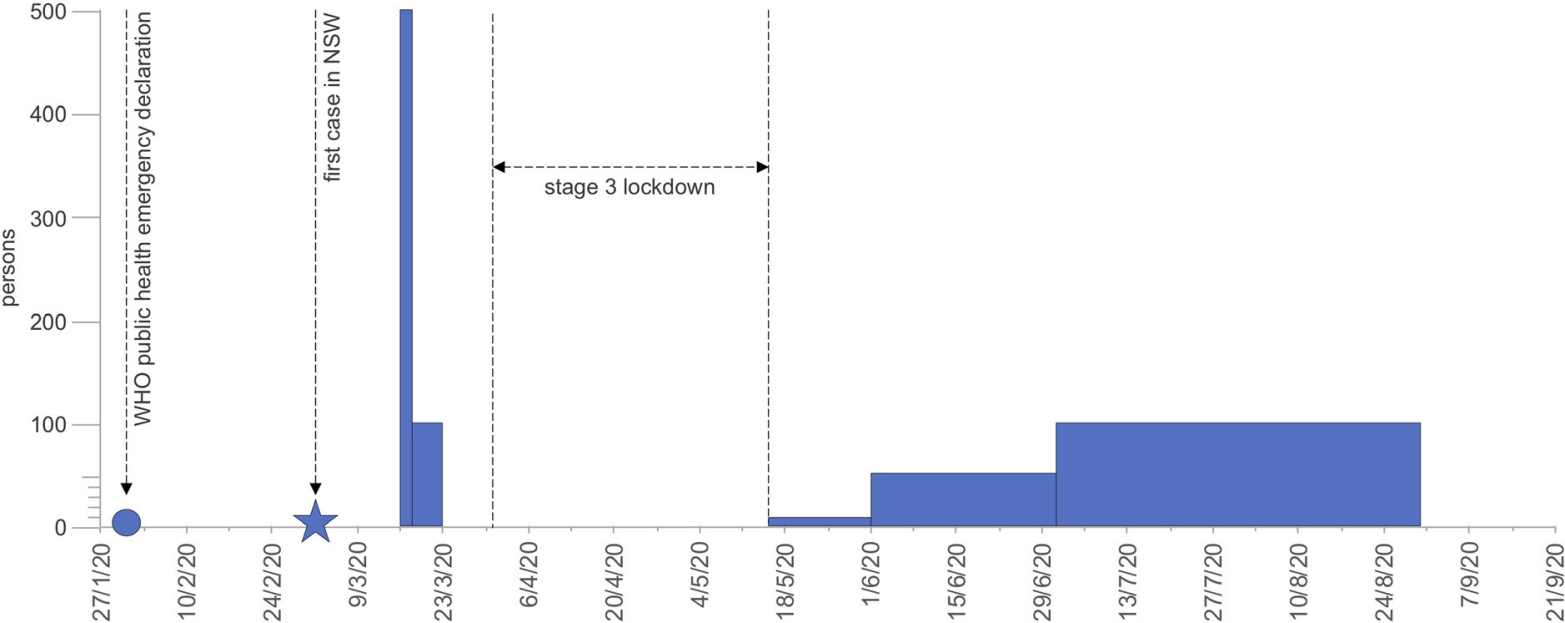
(Color online) Graphic representation of the effects of the public health measures on church services. The bars show the maximum number of attendees as permitted under the regulations. No restrictions prior to 16 March 2020.^1^

Following the first positive case of COVID-19 in NSW on 25 January 2020 and the first case of community transmission on 2 March 2020, the NSW State Government began to implement staged, community-wide public health control plans, placing restrictions on gathering and movement. On 16 March the NSW Minister for Health and Medical Research introduced the first restrictions on gatherings of people to 500 under the Public Health Act. Two days later, the order was amended to restrict public gatherings, including church services, to 500 people outdoors and 100 people indoors. By 23 March, all churches were closed.^1^ Church authorities responded by developing or activating response plans (Fisher, 2020a) that reflected the changing government provisions (Fisher, 2020c) as well as innovative solutions, such as preaching outdoors from the steps of the churches (Patty, 2020). Effective 31 March 2020, all of NSW was under Stage 3 lockdown, with public gatherings limited to two persons, and people were not permitted to leave their place of residence without reasonable excuse. Government imposed restrictions were gradually eased from 15 May 2020 (Fig. 1). Churches immediately followed suit, recommending the recommencement of services for 10 participants as of 15 May (Fisher, 2020d), 50 participants as of 1 June (Fisher, 2020e), and 100 participants as of 2 July 2020 (Fisher, 2020d).

## IV. STATUS OF CHURCH BELL RINGING DUE TO COVID-19

The soundscape generated by church bells depends on the nature of ringing, whether bells are rung as part of liturgical practice, such as calls to prayer or mass, or whether bells are rung as peals, a form of bell music generated by change ringing. While the former requires a single individual to ring the tenor bell, the latter requires a group of bellringers acting in unison, which has obvious implications in times of social distancing rules during a pandemic.

In order to ascertain the impact of the COVID-19 pandemic on church bell ringing, we first sourced denomination-specific directives for NSW churches regarding bell ringing. We then sent a brief targeted questionnaire to church individuals who expressed particular interest from our original 2018 survey, soliciting information pertaining to bell ringing practices during the COVID-19 pandemic, and how ringing patterns have deviated from the norm. We combined these data with information from publicly available sources (including the Australian and New Zealand Association of Bellringers) to present a narrative on the effects (to date) of the pandemic on church bell ringing in NSW.

### A. Bell ringing as part of liturgical practice

In anticipation of the Stage 3 lockdown, the Catholic Archdiocese of Sydney activated its own response plan aimed at ensuring that liturgical services could be provided during lockdown (Fisher, 2020f). The diocese directed that bells be rung five times each day at specific times for prayer (4) and the midday Angelus, “calling on the faithful to unite in a prayer for an end to the COVID-19 pandemic,” with specific reference that “the ringing of the bells will remind the faithful of the importance of pausing and uniting wherever they are in prayer for those suffering due to the coronavirus” (Staff Writer, 2020).

In the early stages of incremental restrictions, the Anglican Cathedrals of Parramatta (population 257 197), Sydney [population 2 085 000 (area of diocese only)] and Wollongong (population 295 669) rang their bells not only on Sunday, but also on Mondays and Thursdays to call for public services and prayer (Davies, 2020), with the services to be delivered from the steps of cathedrals (Patty, 2020), before their termination with the Stage 3 lockdown.

Elsewhere too, bells were used to reaffirm the presence of the church in the community and to provide audible and public emotional support in smaller outer regional communities such as Mudgee (population 10 923 in 2016) (Palmer, 2020), Glen Innes (population 8836) (Messenger, 2020), and Moree (population 7383) (Harris, 2020). This was the case in particular at the time of increasing restrictions, with additional ringing of bells initiated as “a call to action, but also a way for the church to comfort people stuck in quarantine” [David Robinson quoted by Messenger (2020)].

In some instances, this occurred daily, as in the case of the Anglican Churches in Moree (Harris, 2020) and Glen Innes, with the daily ringing at 9 am (Robinson, 2020), and in Mudgee, with the latter ringing its bells seven times at 7 pm (Palmer, 2020). Daily ringing continued at Glen Innes until restrictions lifted, returning to the usual pattern of weekly services and for special occasions (Robinson, 2020). Church bells were also rung on special occasions, such as in support of frontline COVID workers, in places like Mudgee (Palmer, 2020), and for ANZAC Day. Other churches played recordings of their bells, such as St. Matthew's in Albury which played 25 min of change ringing for Victory in the Pacific Day (Jefferies, 2020b).

Other congregations, however, fell silent during lockdown. Some Catholic churches, such as Albury (Wallace, 2020) and Leeton (Murphy, 2020) not only stopped calling mass (as there was none), but also stopped ringing the Angelus. Not surprisingly, it was noted that “[d]uring the lock-down, it was quiet and unusual” (Wallace, 2020). For other churches (e.g., the Anglican church in Albury), the lockdown restrictions meant that manual ringing was prohibited, while technical failures also prevented electronically activated ringing of bells (Jefferies, 2020b).

Once the church services gradually resumed, bell ringing was no longer mentioned in the pastoral letters (Fisher, 2020e,d,b), however, the physical activity of bell ringing continued to be restricted/regulated from 1 July 2020 by the requirement of a COVID-19 safety plan (MHMR, 2020; NSW Health, 2020). While many churches returned to normal services as soon as, or very soon after, regulations permitted (e.g., Catholic and Anglican Dioceses Sydney), in some instances the silence prolonged well past the reduction in restrictions. The Anglican Church in Tamworth, for example, did not resume services and thus bell ringing until mid-August 2020 (146 days of silence) (Bovis, 2020). There are churches that have remained closed for services since the government order of 23 March and have not reopened since. For example, in consequence the tenor bell of the Uniting church in Albury, which until then had been rung every Sunday, has fallen silent (Jones, 2020).

### B. Change ringing (peals)

Imposed regulations also affected the convention of change ringing in many churches. Rapid change of events is clearly evident in St Andrew's Cathedral, Sydney. With cathedral services suspended on 20 March 2020, the usual bell ringing associated with the Sunday services was no longer required, and a 5 min ringing was undertaken from midday for bi-weekly outdoor prayer services instead. Regular weekly evening bell practices were suspended two days later, with training limited to a maximum number of six ringers in the tower, until the cessation of all bell ringing activities on 30 March 2020 (Bosman and Perrins, 2020). Other churches affected include: St Mary's Cathedral, Sydney, where the bells were silenced on 19 March 2020 and “rung down” on 5 April, before an almost two month hiatus, with their return on 7 June with strict rules in place including the operation of the ropes by local members, and single operation of a rope by one individual (Hill, 2020); St Paul's, Burwood, where bell ringing was limited to four members in the tower from 7th June 2020 after an approximately three month hiatus—apart from a tenor bell which tolled regularly for all services at the church (including appropriately physically distanced internal and external services) save for a short period when church services were distributed electronically (Brock, 2020); and Hoskins Uniting Church, Lithgow, where all bell ringing was completely suspended until the creation of a Bellringing Management Plan and subsequent conditional return of limited ringing from July 5 2020 (Musgrave, 2020). Interestingly, the aforementioned plan allows bells to be rung for regular church services only (limited to 6 ringers), but precludes ringing for other occasions, including practice sessions, for visitors, and for weddings or funeral services (Lithgow Bellringers, 2020).

The major inhibitor for a return to full pre-pandemic ringing practices is the public health mandate to (a) maintain a 1.5 m separation between individuals and (b) that any room needs to provide space at a ratio of 4 m^2^ per person. In essence, as most ringing rooms are too small and the ropes are usually less than 1.5 m apart, ringing is either completely suspended (Jefferies, 2020a) or limited to a reduced number of bell ringers (Perrins, 2020). At the height of the pandemic, 64 towers from the Australian and New Zealand Association of Bellringers (ANZAB) had suspended all ringing due to imposed restrictions. On 15 August 2020, 21 NSW towers connected to ANZAB were still suspended, equating to 65.6% of change-ringing, or pealing towers in the state (Australian and New Zealand Association of Bellringers, 2020).

## V. RESULTING CHANGES TO SOUNDSCAPES

**FIG. 2.**
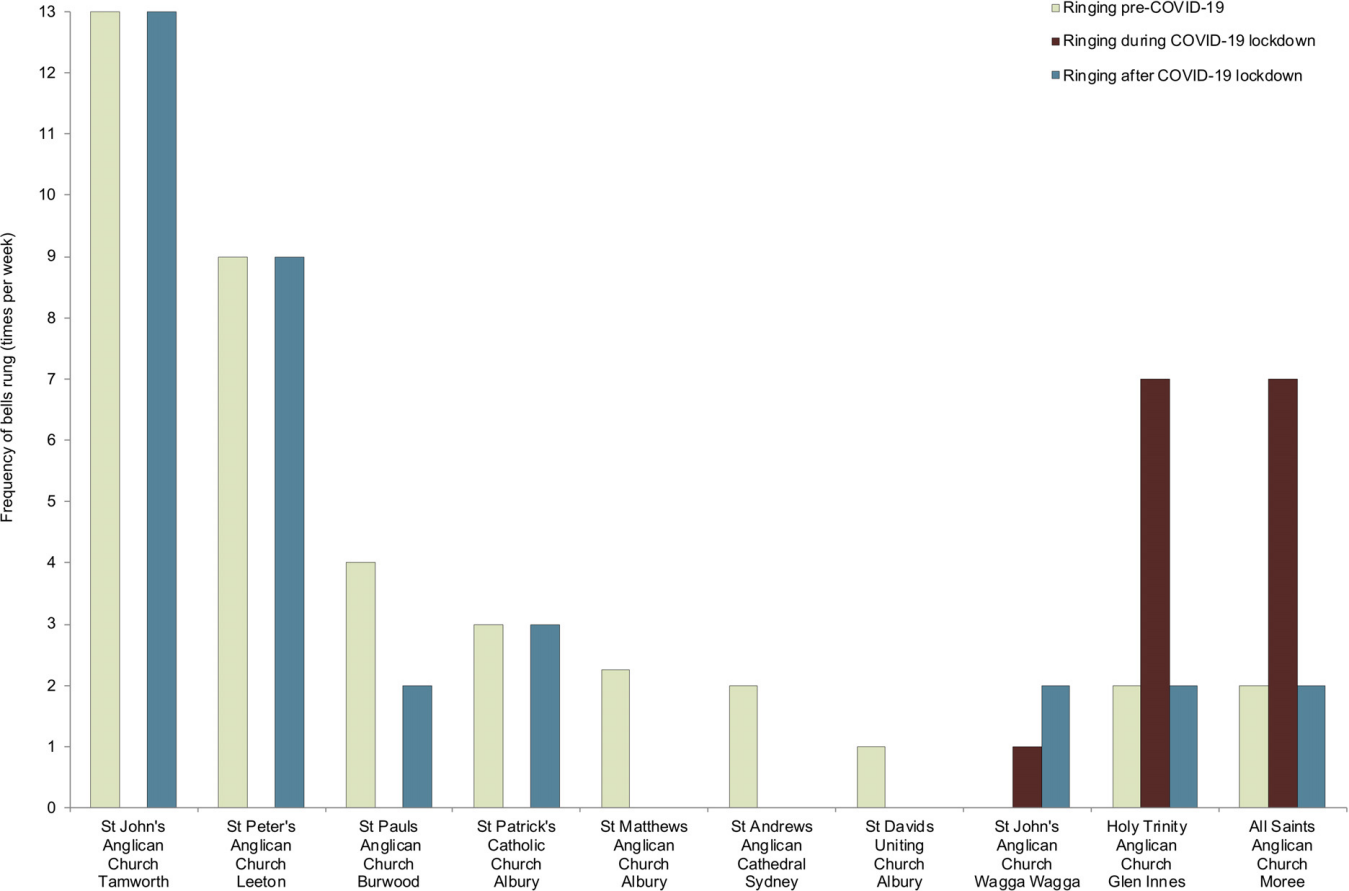
(Color online) Bell ringing patterns of surveyed NSW churches before, during, and after the NSW Stage 3 COVID-19 lockdown.

The changes in church environment soundscapes as a result of the COVID-19 pandemic are not homogenous across regions or denominations. Some churches increased their frequency of bell ringing in communal support for the medical staff and humanity in general, almost in defiance against the difficulties at hand. In cases where churches increased bell ringing, soundscapes here would be richer in this sense, especially in comparison with the expected reduced occurrence of other urban environmental sounds as a result of movement restrictions. Such an example would be the soundscape of Glen Innes, with a seven-fold increase in bell ringing at the Anglican church, coinciding with regular ringing of the Catholic church (twice daily) and the Uniting church (weekly) (Robinson, 2020) (Fig. 2). On the opposite end of the spectrum were churches that fell totally or largely silent during the lockdown period until they recommenced church services, either because it was unnecessary or unfeasible to ring bells, resulting in a reduced richness of soundscape. This applied, for example, to Anglican and Uniting churches in inner and outer regional areas such as Leeton (population 11 168), Tamworth (population 42 872) and Albury (population 54 477). In Leeton, the typical bi-weekly broadcast of recorded bells of St Peter's Anglican Church ceased for 70 days while the church was closed, apart from one calling at Easter (Murphy, 2020). Setting aside additional ringing for weddings and funerals, St John The Evangelist Anglican Church, Tamworth, rang the bells thirteen times a week prior to COVID-19, with Saturday the only day the bells could not be heard (Bovis, 2020). For the next twenty weeks its bells were silent. The resulting soundscape was likewise heavily affected at St Andrew's Cathedral, Sydney, where not only the frequency of bell ringing was decreased (from 2.5 h to five minutes per week), but also with a reduced intensity of the sound source and subsequent sound richness (from 12 bells proficiently rung in tandem to a solitary bell rung by an office worker) (Perrins, 2020). In some communities the change in soundscape was profound. Prior to the COVID lockdown, the Catholic Church in Albury had rung the Angelus daily at noon (Wallace, 2020), the Anglican church had rung the Angelus three times each day and its peals at least twice a week (training and Sundays) (Jefferies, 2020a; MacLeod-Miller, 2020), and the Albury Uniting Church its tenor bell every Sunday (Jones, 2020). During lockdown the Albury soundscape was devoid of any church bell ringing sounds, and since lockdown ended, the only church bell was that of the Catholic Angelus being rung only on Sundays.

There was a distinct lack of bells rung for weddings during the COVID-19 pandemic, and this correlates with the lack of church-based weddings over this time. Whilst this is expected to partially correlate with imposed restrictions on the numbers permitted at weddings in NSW, many churches reported that the period of June-August (winter) was also generally poorly represented in 2019. It would be interesting to see how bells are represented for this activity and the resultant soundscape in NSW as Australia approaches spring and into summer.

## VI. CONCLUSION

The COVID-19 pandemic has demonstrated how a stochastic disruptive event can dramatically alter community soundscapes. With the reduction in ambient transportation noises due to the reduction in human activity during the lockdown period, the ringing of church bells created a stronger audible presence than otherwise. Likewise, the absence of church bells sounds made the COVID-19 silence even more pronounced.

Unlike soundscapes that are a by-product of human activity and thus sensitive to changes in that activity (e.g., aircraft and traffic sounds), church bell ringing creates a soundscape that is directly influenced by purposive human action. We have shown elsewhere the myriad of values imparted by society on church bell ringing in NSW, being both religious and secular in origin (Parker and Spennemann, 2020).

The COVID-19 induced changes to urban soundscapes have brought to the fore the role and power of the individual. Some clergy acquiesced to the situation and embraced the physical shutting down of services as new reality, pausing all church bell sounds in the process. For others, however, COVID-19 enkindled a sense of defiance, whereby the ringing of the bells was construed as a public signaling system to alert the community of the continued presence of the church.

It remains to be seen to what extent the pandemic engenders a “new normal,” with durably altered soundscapes, or whether old patterns reemerge once the community has put the pandemic behind it.

## Footnotes

^1^ See supplementary material at https://www.scitation.org/doi/suppl/10.1121/10.0002451 for 2018 data on the ringing of church bells in NSW and for details of the development of COVID-19 related public health orders issued by NSW.
